# Estrogen-dependent DLL1-mediated Notch signaling promotes luminal breast cancer

**DOI:** 10.1038/s41388-018-0562-z

**Published:** 2018-11-15

**Authors:** Sushil Kumar, Ratnesh Kumar Srivastav, David W. Wilkes, Taylor Ross, Sabrina Kim, Jules Kowalski, Srinivas Chatla, Qing Zhang, Anupma Nayak, Manti Guha, Serge Y. Fuchs, Christoforos Thomas, Rumela Chakrabarti

**Affiliations:** 1 Department of Biomedical Sciences, School of Veterinary Medicine, University of Pennsylvania, Philadelphia, PA 19104 USA; 2 Department of Pathology and Laboratory Medicine, Lineberger Comprehensive Cancer Center, University of North Carolina at Chapel Hill, Chapel Hill, NC 27599 USA; 3 Department of Pathology and Laboratory Medicine at the Hospital of the University of Pennsylvania, University of Pennsylvania Perelman School of Medicine, Philadelphia, PA 19104 USA; 4 Department of Radiation Oncology, University of Pennsylvania Perelman School of Medicine, Philadelphia, PA 19104 USA

**Keywords:** Breast cancer, Oncogenes

## Abstract

Aberrant Notch signaling is implicated in several cancers, including breast cancer. However, the mechanistic details of the specific receptors and function of ligand-mediated Notch signaling that promote breast cancer remains elusive. In our studies we show that DLL1, a Notch signaling ligand, is significantly overexpressed in ERα^+^ luminal breast cancer. Intriguingly, DLL1 overexpression correlates with poor prognosis in ERα^+^ luminal breast cancer, but not in other subtypes of breast cancer. In addition, this effect is specific to DLL1, as other Notch ligands (DLL3, JAGGED1, and JAGGED2) do not influence the clinical outcome of ERα^+^ patients. Genetic studies show that DLL1-mediated Notch signaling in breast cancer is important for tumor cell proliferation, angiogenesis, and cancer stem cell function. Consistent with prognostic clinical data, we found the tumor-promoting function of DLL1 is exclusive to ERα^+^ luminal breast cancer, as loss of DLL1 inhibits both tumor growth and lung metastasis of luminal breast cancer. Importantly, we find that estrogen signaling stabilizes DLL1 protein by preventing its proteasomal and lysososmal degradations. Moreover, estrogen inhibits ubiquitination of DLL1. Together, our results highlight an unexpected and novel subtype-specific function of DLL1 in promoting luminal breast cancer that is regulated by estrogen signaling. Our studies also emphasize the critical role of assessing subtype-specific mechanisms driving tumor growth and metastasis to generate effective subtype-specific therapeutics.

## Introduction

Breast cancer is the second leading cause of cancer-related death among women worldwide. The heterogeneity and diversity of breast cancer challenges both the choice and efficacy of treatment options, thereby enhancing the mortality of breast cancer patients [1, 2]. The high heterogeneity of breast cancer is primarily due to the presence of several molecular subtypes that can be distinguished by the expression of estrogen receptor (ERα), progesterone receptor (PR) and human epidermal growth factor receptor 2 (HER2). These subtypes include luminal A and luminal B (ERα^+^ and PR^+^), basal like/Triple negative breast cancer (TNBC, which lacks ERα and PR and HER2) and HER2^+^ [3]. Recent report indicates that clinical outcome and/or survival notably differs between these subtypes [4], highlighting the need to understand breast cancer cell signaling in a subtype-specific manner for better stratification of patients and optimal development of targeted therapeutic options.

The most common subtype of breast cancer is the Luminal ERα^+^ subtype (Luminal A and Luminal B), which makes up 80% of all breast cancer. Estrogen drives the growth of ERα^+^ breast tumors by stimulating both survival and proliferation of breast cancer cells [5–7]. Current treatment options for luminal tumors include surgery, chemotherapy, radiotherapy, and anti-estrogen therapy [8]. While initial treatment can be promising, tumor recurrence and metastasis are a main cause of death in these patients. Understanding the molecular mechanisms for tumor growth and metastasis of estrogen-responsive luminal tumors should lead to identification of new molecules for targeted therapy that would improve the prognosis for this large subset of patients.

The Notch signaling pathway is an evolutionarily conserved receptor-ligand-based system operated by four Notch receptors (Notch1–4) and five ligands (Jag1, Jag2, Dll1, Dll3, and Dll4) of the Jagged/Serrate and Delta families respectively [9, 10]. Notch signaling relies on cell−cell contact and regulates various developmental and homeostatic processes [11–14]. The biological function of Notch signaling in various cancers has been studied [15, 16] and its deregulation promotes breast cancer [17, 18], suggesting that targeting Notch signaling could be a promising strategy for the development of breast cancer therapeutics [19, 20]. Inhibition of Notch signaling can be achieved at different levels by either small molecule inhibitors or monoclonal antibodies that block the Notch receptors. Gamma-secretase inhibitors (GSIs) inhibit Notch signaling and effectively repress cancer stem cells and, therefore, breast cancer growth [21, 22]. Unfortunately, GSIs show various off-target effects in patients [23, 24] and deleterious GSI-induced side effects in the gastrointestinal tract [25], limiting its therapeutic benefits. These studies highlight the need for a more specific receptor and/or ligand-based therapy as a potentially safer and more effective therapeutic option.

Most studies assessing the effects of Notch signaling in breast cancer have focused on the receptors. Although elevated expression of the Jagged1 (Jag1) ligand is associated with both poor prognosis and bone metastasis in breast cancer patients [17, 26], a detailed analysis of the functional role of Notch ligands in breast cancer initiation, growth and metastasis is still lacking. However, we have recently shown that Dll1, an active Notch ligand that belongs to the Delta family and is implicated in intestinal functions [27], is also expressed in mammary stem cells and interacts with stromal macrophages to maintain the stem cell niche in normal mammary gland [28]. Dll1 has also been implicated in promoting tumor initiating cells/cancer stem cells in multiple cancers such as glioblastoma, renal cell carcinoma, and rhabdomyosarcoma [29–31] and enhancing T-cell-mediated antitumor immunity [32]. However, the function of Dll1 in breast cancer remains elusive.

Using primary patient breast tumors, we show here for the first time that DLL1 protein expression is highly upregulated in ERα^+^ luminal breast cancer compared to either normal breast tissue or TNBC/basal breast cancer. In addition, high *DLL1* expression (*DLL1*^high^) correlates with decreased distant metastasis-free survival (DMFS) of ERα^+^ luminal breast cancer patients. Moreover, DLL1^high^ protein expression correlates with poor overall survival of ERα^+^ luminal breast cancer patients. Intriguingly, *DLL1* expression levels in ERα^−^ subtypes of breast cancer, including TNBC/basal and HER2^+^, do not correlate with prognosis, highlighting a potential subtype-specific function for DLL1 in ERα^+^ breast cancer. In support, knockdown of DLL1 in ERα^+^ luminal breast cancer cells reduces primary tumor growth and metastasis in ERα^+^ tumors, but not in tumors of the TNBC/basal subtype. Loss of DLL1 inhibits several essential processes of breast cancer, including proliferation, maintenance of breast cancer stem cell number, and angiogenesis. Finally, overexpression of Dll1 leads to more tumor growth and increased metastasis, confirming that DLL1 expression strongly influences the growth of primary tumors and metastasis in ERα^+^ luminal breast cancer. Mechanistically, we show that ERα-signaling stabilizes DLL1 protein levels by reducing proteasomal and lysosomal degradation. We further demonstrate that the Dll1 protein is ubiquitinated in the absence of hormones such as estrogen, suggesting that ERα-signaling inhibits ubiquitination of DLL1, thereby reducing proteasomal degradation. Together, our data demonstrate a novel tumor-promoting function for the Notch ligand, DLL1 in ERα^+^ luminal breast cancers, thereby providing initial proof-of-principle for subtype-specific therapies for luminal ERα^+^ breast cancer patients.

## Results

### DLL1 is overexpressed and is associated with poor prognosis in luminal breast cancer patients

**Fig. 1:**
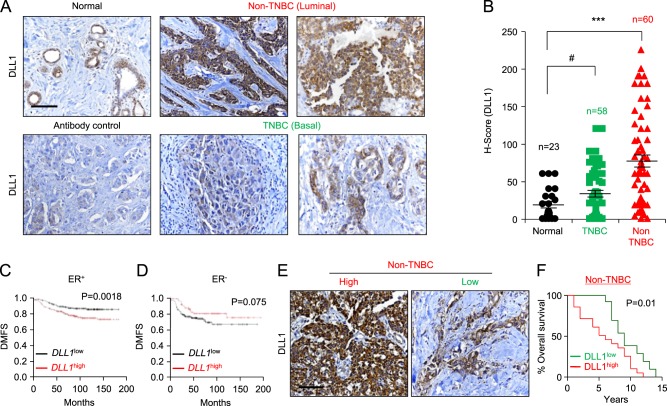
DLL1 expression is higher in luminal (non-TNBC) breast cancer patients and is associated with poor patient survival. **a**, **b** Representative IHC images (**a**) and calculated H-score (**b**) show higher DLL1 protein levels in non-TNBC (*n* = 60) compared to tumor-adjacent normal tissue (*n* = 23) and TNBC (*n* = 58) patient tumors. The H-Score was calculated by multiplying intensity with abundance. Negative control is tumor tissue stained with anti-IgG antibody showing no nonspecific staining. **c**, **d** Kaplan–Meier (KM) plots show poor distant metastasis-free survival (DMFS) of breast cancer patients by *DLL1* expression status (*DLL1*^high^ or *DLL1*^low^) in ER^+^ (**c**), ER^−^ (**d**). *n* = 965 ER^+^ patients (**c**) and *n* = 430 ER^−^ (**d**) patients were used to make KM-plots. **e** Representative IHC images of non-TNBC patient breast tumors show DLL1^high^ and DLL1^low^ protein expression respectively. **f** KM plot shows poor patient survival of patients with high levels of DLL1 protein compared to patients with low levels of DLL1 protein. H-score was evaluated to stratify patients into DLL1 high and low expressers based on the IHC with DLL1 antibody on the non-TNBC patient breast tumors. *n* = 32 non-TNBC samples were used with overall survival data. Samples were stratified into DLL1^low^ (*n* = 12) and DLL1^high^ (*n* = 20) groups for KM plot analysis. **b** Mann−Whitney *U* test and **c**, **d**, **f** Log-rank test was used to calculate *p* values. **b** Data are presented as the mean ± SEM. ***p < 0.001 and # nonsignificant. Scale bars, 40 µm (**a**, **e**)

To investigate the clinical significance of DLL1 in breast cancer, we assessed DLL1 protein expression by performing IHC on primary human patient samples (TNBC patients *n* = 58, non-TNBC patients *n* = 60, and adjacent normal tissue *n* = 23) using an anti-DLL1 antibody (Fig. 1a, b and Supplementary Table S1A). We scored the intensity of DLL1 protein expression (0–3) and abundance (0–100) of DLL1^+^ tumor cells. The intensity and abundance were multiplied to get a H-score (0–300). The specificity of the anti-DLL1 antibody was validated by western blot of human breast cancer cell lines, which show a single band at the expected size of ~70 kDa (Supplementary Fig. S1A). Our IHC analyses revealed significantly higher levels of DLL1 protein expression in the luminal/non-TNBC subtype compared to either normal cells or TNBC/basal breast cancer samples (*p* < 0.0001) (Fig. 1a, b). The modest difference in DLL1 expression between normal tissue and TNBC was not statistically significant (*p* = 0.062), suggesting that DLL1 is predominantly upregulated in non-TNBC/luminal patient samples. The clinical correlation data using published datasets were further confirmed by western blot analysis of human breast cancer cells that showed that DLL1 expression is higher in three luminal breast cancer cells (MCF7, T47D and ZR-75-1). In contrast, DLL1 expression is observed in only three (SUM159, Hs578t, and HCC1806) of five TNBC cell lines and at its expression was at a lower level compared to luminal cells (Supplementary Fig. S1A).

To determine if DLL1 expression was associated with disease progression, we tested the correlation between DLL1 expression (high and low) and DMFS using published Kaplan–Meier (KM) plotter datasets [33]. We found that *DLL1*^high^ ER^+^ luminal breast cancer patients have poor clinical outcome compared to *DLL1*^low^ patients (Fig. 1c). Intriguingly, there was a trend for ER^−^ basal tumors to display the opposite result, with *DLL1*^high^ patients having a better clinical outcome compared to *DLL1*^*l*ow^ patients (Fig. 1d). Furthermore, when *DLL1* expression was compared with DMFS in four different molecular subtypes of breast cancer, higher *DLL1* levels strongly correlated with poor patient outcome in the ERα^+^ Luminal A subtype, but not in the ERα^low^ subtypes such as luminal B, TNBC/basal, and HER2 (Supplementary Fig. S1B-E). A modest (yet not statistically significant) trend was observed in Luminal B breast cancer patients. *DLL1* expression tended to correlate with increased DMFS in the basal subtype, similar to what was observed for the ERα^–^ subtype (Supplementary Fig. S1D). To determine if *DLL1* played a predominant role in Notch signaling in ER^+^ subtypes, additional Notch ligands were evaluated. We found that high expression of *DLL1* showed the strongest positive correlation with poor patient outcome (*p* = 0.0018) among all Notch ligands, although *DLL4* (*p* = 0.03) showed a similar correlation to *DLL1* (Fig. 1c and Supplementary Fig. S1F-I). To test if DLL1 protein levels also correlate with overall survival of non-TNBC/luminal ER^+^ patients, patient samples (*n* = 32 non-TNBC and *n* = 21 for TNBC) were obtained with overall survival data (Supplementary Table S1B). We categorized ERα^+^ luminal patient samples into DLL1^high^ and DLL1^low^ groups on the basis of H-score derived from patient breast tumors stained with DLL1 antibody. We found that DLL1^high^ patients have a significantly poorer overall survival compared to DLL1^low^ patients in the non-TNBC/luminal subset, but not in TNBC patients (Fig. 1e, f and Supplementary Fig. S1J-K). Altogether, our data suggest a protumor function of Notch ligand DLL1 in non-TNBC/luminal breast cancer, which is associated with worse prognosis/survival in the patient samples.

### DLL1 plays a subtype-specific function in primary tumor growth, progression, and metastasis in breast cancer

**Fig. 2:**
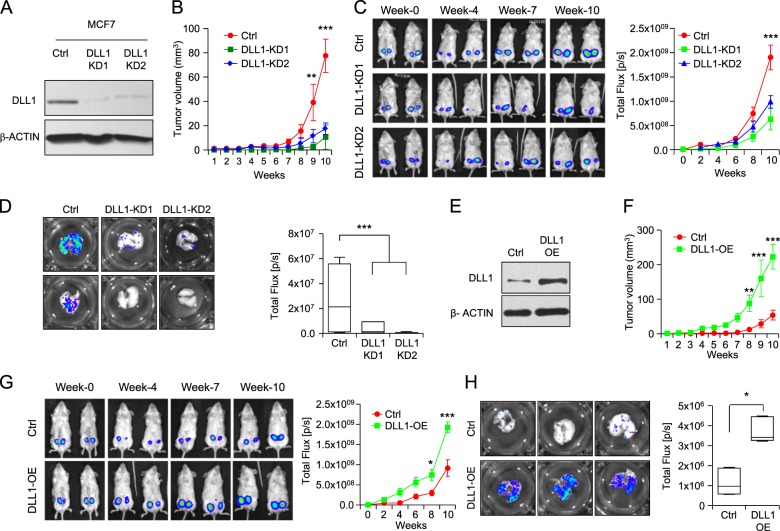
DLL1 promotes human ERα^+^ luminal tumor growth and metastasis. **a** Western blot shows DLL1 protein expression in MCF7 control and DLL1-KD (KD1 and KD2) cells after lentiviral shRNA-mediated knockdown (KD). Firefly luciferase-expressing MCF7 control and DLL1-KD (KD1 and KD2) cells (2×10^6^) were injected into mammary fat pad (MFP) of NSG mice. **b** Tumor growth curves show tumor volume of indicated groups. Representative mice images (**c**, left) and tumor growth curves (**c**, right) (total flux, photons per second, p/s) show bioluminescent signal from tumors in vivo. **b**, **c** *n* = 8 tumors/group. Contralateral mammary glands (fourth position) of *n* = 4 mice were used for injection/group. **d** Ex vivo lung metastasis images from mice injected with MCF7 control and DLL1-KD (KD1 and KD2) cells using bioluminescence imaging (BLI). **d** *n* = 4 mice/group. **e** Western blot show DLL1 protein expression in MCF7 control and DLL1-OE cells after lentivirus-mediated overexpression (OE) of DLL1. Firefly luciferase-expressing MCF7 control and DLL1-OE cells (2×10^6^) were injected into mammary fat pad (MFP) of NSG mice. **f** Tumor growth curves show palpated data of tumor volume of indicated groups. Representative mice images (**g**, left) and tumor growth curves (**g**, right) (total flux, photons per second, p/s) show bioluminescent signal from tumors in vivo. **f**, **g** *n* = 6 tumors/group. Contralateral mammary glands (fourth position) of *n* = 3 mice were used for injection/group. **h** Ex vivo BLI images of lungs from mice injected with MCF7 control and DLL1-OE cells show metastases in indicated groups. *n* = 3 mice/group. **d**, **h** Mann−Whitney *U* test were used to compute *p* value. **b**, **c**, **f**, **g** Two-way ANOVA test with Bonferroni correction was performed to compute statistical significance for tumor growth curve data. Data are presented as the mean ± SEM. *p < 0.05, **p < 0.01 and ***p < 0.001

To understand the functional role of DLL1 in growth and metastasis of human breast cancer (luminal and TNBC), we used the ERα^+^ luminal cell lines MCF7 and T47D, and ER^−^ basal/TNBC cell line, HCC1806 [34, 35]. MCF7 and HCC1806 were engineered to express firefly luciferase and red fluorescent protein (RFP) to facilitate in vivo tracking, respectively [35]. To knockdown human DLL1 in these cancer cells, we utilized shRNA/lentivirus approach. After evaluating five shRNA clones (Supplementary Fig. S2A), we chose two shRNA clones to continue further investigation. DLL1 was successfully knocked down at both the protein and mRNA levels in luminal MCF7 cells using these shRNAs (Fig. 2a and data not shown). Both MCF7 DLL1 knockdowns (KD1 and KD2) exhibited reduced primary tumor growth in NSG mice (Fig. 2b, c) compared to control cells (that received an empty shRNA vector), corroborating observations in human patients with ER^+^ breast cancer (Fig. 1). We made a similar observation with another luminal cell line T47D, demonstrating that DLL1 knockdown tumor cells (KD1 and KD2) had slower growth compared to control (Supplementary Fig. S2B-C).

To determine if DLL1-deficiency in tumor cells affects lung metastasis, we harvested lungs and acquired bioluminescence signals (BLI). Notably, MCF7 DLL1-KDs had significantly reduced BLI signals in the lungs compared to control, indicating fewer tumor cells in the metastatic site (Fig. 2d and Supplementary Fig. S3A). These data were consistent with clinical data demonstrating increased DMFS and survival in DLL1^low^ ER^+^ luminal patients (Fig. 1c, e, f). In complementary experiments, we next tested if increasing DLL1 expression in luminal cells further augments tumor growth and metastasis. For this, we again used a lentiviral approach to establish MCF7 cells stably overexpressing DLL1 (DLL1-OE) (Fig. 2e). Tumor growth was significantly enhanced in MCF7 DLL1-OE compared to empty vector control (Fig. 2f, g). Furthermore, we found higher BLI signals in the lungs, demonstrating increased metastasis in mice injected with DLL1-OE MCF7 cells compared to control (Fig. 2h and Supplementary Fig. S3B).

**Fig. 3:**
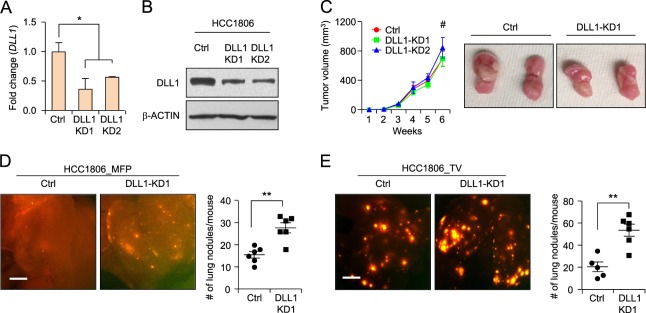
DLL1 does not influence tumor growth but inhibits metastasis in human TNBC. **a, b** qPCR and western blot data show DLL1 mRNA and protein levels in human TNBC cell line HCC1806 after lentivirus-mediated knockdown (KD) of DLL1. **c** 200,000 HCC1806 control and DLL1-KDs (KD1 and KD2) cells were injected into mammary fat pad of NSG mice. Tumor growth curves (**c**, left) and representative whole tumor images (**c**, right) show no significant difference in growth of HCC1806 DLL1-KDs (KD1 and KD2) primary tumors compared to control, *n* = 6 mice used per group. **d** Representative whole mount images of lungs from mice with mammary fat pad injection (MFP) show metastasis as seen by RFP positivity (**d**, left) and respective quantification is shown in (**d**, right). **e** 200,000 HCC1806 control and DLL1-KD1 tumor cells were injected into blood stream of NSG mice through tail-vein. Lung metastasis as seen by RFP^+^ nodules show higher number of RFP^+^ lung nodules in DLL1-KD1 compared to control (**e**, left). Quantification is shown in (**e**, right). *n* = 5 mice (Ctrl) and *n* = 6 mice (DLL1-KD1) were used. **d**, **e** Mann−Whitney *U* test and **c** two-way ANOVA test with Bonferroni correction was performed to compute statistical significance. Scale bars, 500 µm in (**d**, **e**). **a** Data are presented as the mean ± SD. **c−e** Data are presented as the mean ± SEM. *p < 0.05, **p < 0.01 and #nonsignificant

To determine the function of DLL1 in human TNBC cells, we made DLL1-KD HCC1806 cell lines with two shRNA clones and confirmed the reduction in DLL1 mRNA and protein levels compared to control (Fig. 3a, b). There was no significant difference observed in tumor growth with decreased DLL1 expression in HCC1806 cells compared to control (Fig. 3c). However, reduction of DLL1 leads to increased metastasis in DLL1-KD1 HCC1806 compared to control (Fig. 3d), which was further confirmed by injecting tumor cells using a tail-vein metastasis assay (Fig. 3e). Taken together, our data suggest that while DLL1 promotes ERα^+^ luminal cell tumor growth and metastasis and it has no significant effect on the TNBC tumor growth.

### Loss of Dll1 reduces primary tumor growth and metastasis in mouse luminal breast cancer cells

**Fig. 4:**
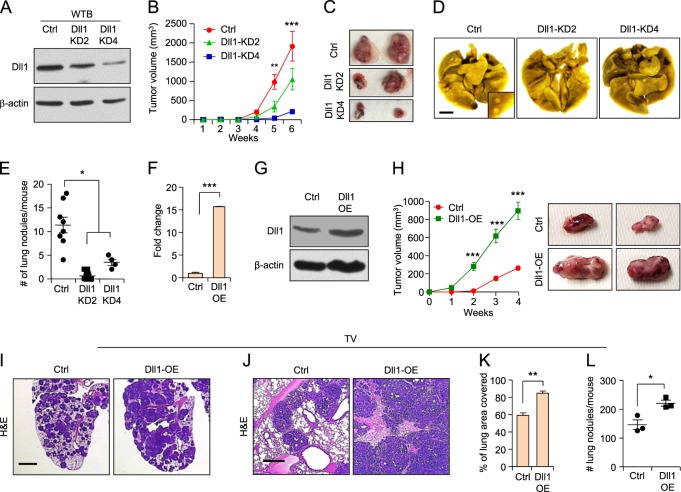
Dll1 promotes tumor growth and metastasis in mouse luminal/non-TNBC tumors. **a** Western blot data showing Dll1 protein expression in control and Dll1-KD2 and Dll1-KD4 WTB luminal cells after lentiviral shRNA-mediated knockdown of WTB cells (KD). **b**, **c** WTB control and Dll1-KD (KD2 and KD4) cells (200,000 cells/injection) were injected into mammary fat pad of FVB mice, and tumor growth was observed by weekly palpation. Representative tumor growth curves (**b**) and whole tumor images (**c**) show reduced tumor growth of WTB Dll1-KD (KD2 and KD4) cells compared to control. **d**, **e** Representative lung images show metastatic lung nodules in (**d**) and respective quantification of lung nodules in (**e**) in mice after mammary fat pad injection (MFP). **b**−**e** Ctrl *n* = 8, Dll1-KD2 *n* = 7 and Dll1-KD4 *n* = 4 mice. **f**, **g** qPCR and western blot data showing Dll1 mRNA and protein expression in control and Dll1-OE WTB luminal cells after lentiviral-mediated overexpression of Dll1 (OE). **h** WTB control and Dll1-OE cells (200,000 cells/injection) were injected into mammary fat pad of FVB mice, and tumor growth was observed by weekly palpation. Representative tumor growth curves (**h**, left) and whole tumor images (**h**, right) show enhanced tumor growth of WTB Dll1-OE cells compared to control. **i−l** 500,000 WTB cells were injected into blood stream of FVB mice through tail-vein. **i**, **j** Representative H&E lung images at low and high magnifications show higher number of lung nodules in lungs of mice injected with WTB Dll1-OE cells compared to control. **k**, **l** Quantification of area and number of lung metastatic nodules of indicated groups. **h−l** *n* = 3 mice/group. Scatter plots represent number of animals as dots (*n* = 3). **e**, **f**, **k** and **l** Mann−Whitney *U* test and **b**, **h** two-way ANOVA test with Bonferroni correction was performed to compute statistical significance. Scale bars, 500 µm (**d**), 200 µm (**i**) and 100 µm (**j**). **f** Data are presented as the mean ± SD. **b**, **e**, **h**, **k−l** Data are presented as the mean ± SEM. *p < 0.05, **p < 0.01 and ***p < 0.001

Since the immune system is an important determinant of the tumor microenvironment, dictating tumor growth and metastasis [36, 37], we further extended our study to a syngeneic mouse breast cancer cell model. Mouse ERα^+^ luminal breast cancer cell line WTB was derived from the MMTV-PyMT luminal tumor model [38], which closely mimics human luminal tumor growth and metastasis [39]. WTB cells show higher expression of luminal markers such as estrogen and progesterone receptors (ERα and PR) compared to the TNBC cell line (4T1) (Supplementary Fig. S4A, B). We stably knocked down Dll1 using lentivirus-mediated shRNA clones in WTB cells. As above, five shRNA clones were tested and two were chosen for functional studies (Supplementary Fig. S2D). Both WTB Dll1-KD clones showed a significant reduction in Dll1 expression at the mRNA and protein levels (Fig. 4a and data not shown). Similar to our results in human luminal cell lines, we found a severe reduction in tumor growth (Fig. 4b, c) from Dll1-KD2 and Dll1-KD4 WTB cells compared to control (Fig. 2). To assess metastasis, we injected tumor cells utilizing the tail-vein method. Notably, both WTB Dll1-KD2 and Dll1-KD4 cells showed a greatly reduced number of lung metastatic nodules compared to control cells (Fig. 4d, e), suggesting an important role for Dll1 in metastatic colonization. As in the human MCF7 luminal cell line (Fig. 2), we found that overexpression of Dll1 leads to increased tumor growth of WTB cells (Fig. 4f–h). Evaluation of the number and area of lung metastasis in lungs of mice injected with control and Dll1-KD tumor cells further confirmed the prometastatic function of Dll1 in WTB luminal cells (Fig. 4i−l). In contrast to mouse luminal tumors, reduced levels of Dll1 in mouse TNBC (4T1) cells show no major difference in tumor growth and metastasis compared to control (Supplementary Fig. S5A–E). Taken together, our data strongly indicate a unique function of Dll1 in promoting multiple steps of cancer growth, progression, and lung metastasis in luminal breast cancer. Thus, our studies highlight the critical role of assessing subtype-specific mechanisms driving tumor growth and metastasis to generate effective subtype-specific therapeutics.

### Dll1 is important for cell proliferation and angiogenesis in luminal breast cancer

**Fig. 5:**
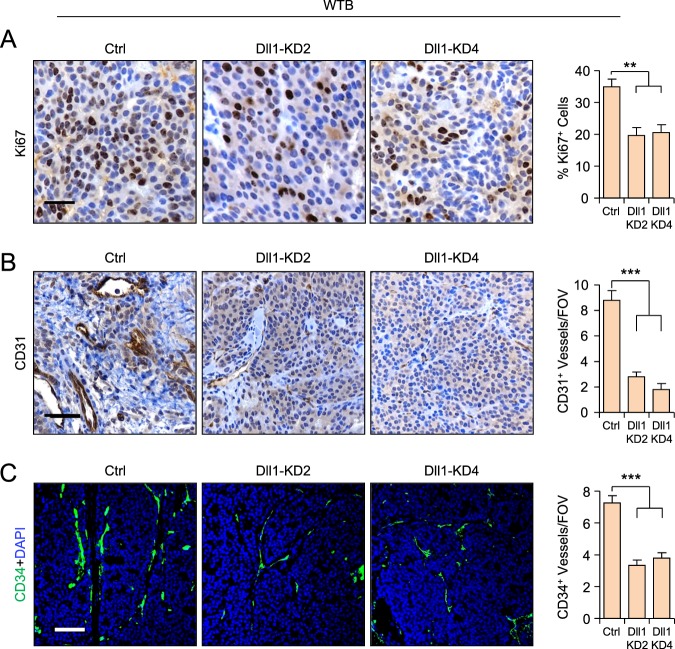
Reduction of Dll1 in mouse luminal tumors leads to decreased proliferation and angiogenesis. **a, b** Representative IHC images show reduced proliferation (Ki67^+^ cells) (**a**) and CD31^+^ blood vessels (**b**) in WTB Dll1-KD (KD2 and KD4) primary tumors compared to control. **c** Representative IF images show reduced CD34^+^ blood vessels in WTB Dll1-KDs (KD2 and KD4) primary tumors compared to control. Quantification is shown in the right of each set of images. **a−c** Mann−Whitney *U* test to compute *p* values. Scale bars, 40 µm (**a−c**). Data are presented as the mean ± SEM. **p < 0.01 and ***p < 0.001. IHC images were quantified from ten random fields and three different samples per group were used. FOV field of view

To delineate how Dll1 promotes luminal tumor growth and progression, we assessed Ki67 expression, a marker of cell proliferation, in MCF7 and WTB tumor sections. We found that both WTB and MCF7 Dll1-KD tumors showed a significant decrease in Ki67^+^ cells compared to control tumors (Fig. 5a and supplementary Fig. S6A), suggesting that decreased proliferation accounts in part for the slower tumor growth. As the Notch ligands Jagged1 and Dll4 promote angiogenesis in several cancers, including breast cancer [40, 41] and increased angiogenesis is associated with increased metastasis in cancer [42], we next assessed the expression of CD31 and CD34, both of which are used to measure angiogeneic blood vessels in tumors. We observed a significantly reduced number of CD31^+^ and CD34^+^ blood vessels in WTB and MCF7 Dll1-KD tumors compared to control tumors, suggesting a possible role for Dll1 in angiogenesis in luminal tumors (Fig. 5b, c and supplementary Fig. S6B, C). Thus, Dll1 may enhance both proliferation and angiogenesis to promote tumor formation and progression of ERα^+^ luminal breast cancer.

### Loss of Dll1 reduces the cancer stem cell population of luminal tumors

**Fig. 6:**
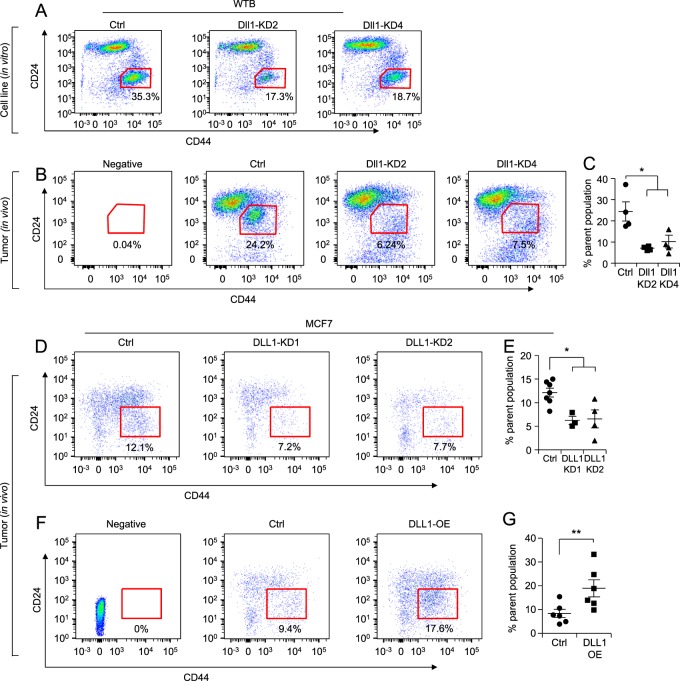
Dll1 promotes cancer stem cell (CSC) population in luminal/non-TNBC tumors. **a** FACS data show reduced in vitro CSC population (CD24^−^CD44^+^ population) in WTB Dll1-KDs (KD2 and KD4) cells compared to control cells. **b** FACS data show reduced in vivo CSCs population in primary tumors derived from injection of WTB Dll1-KDs (KD2 and KD4) cells compared to control tumors using CD24/CD44 markers. **c** Bar graph shows percentage of CSC population in WTB Dll1-KD (KD2 and KD4) tumor cells compared to control tumor cells. **d**, **f** FACS data show reduced in vivo CSC population (CD24^−^CD44^+^ population) in primary tumors derived from injection of MCF7 DLL1-KD (KD1 and KD2) cells and DLL1 overexpression (DLL1-OE) cells compared to their respective control tumors. **e**, **g** Bar graphs show the quantification of CSC population (CD24^−^CD44^+^ population) in DLL1-KDs (KD1 and KD2) and DLL1-OE compared to their respective controls. Scatter plots represent number of animals as dots. Mann−Whitney *U* test was used to compute *p* values. Data are presented as the mean ± SEM. *p < 0.05 and **p < 0.01

Notch signaling promotes cancer stem cell function in many cancers, including breast cancer [43]. As metastasis is associated with increased cancer stem-like cells that is associated with increased dissemination of cells from the primary tumor site to distant metastatic sites [44], we next assessed the role of DLL1 in these additional functions in luminal breast cancer. To assess the tumor-initiating cells/cancer stem cell (TIC/CSC) population, we undertook a flow cytometric analysis using standard CSC markers such as CD24 and CD44^11, 12^ and CD90 [45] (Fig. 6, Supplementary Fig. S7A-C). CSCs (CD24^−^CD44^+^ and CD90^+^) were significantly reduced in WTB Dll1-KDs (DLL1-KD2 and DLL1-KD4) cells compared to control (Fig. 6a and Supplementary Fig. S7C). Similar findings were made in vivo growing tumors (Fig. 6b, c). We observed a similar reduction in CSCs in human MCF7 luminal cells with loss of DLL1 (Fig. 6d, e). Notably, DLL1-OE tumors (in vivo) showed significantly higher numbers of CSCs (CD24^−^CD44^+^) compared to the control (Fig. 6f, g), suggesting an important role for DLL1 in maintaining the CSC population. Accordingly, tumorsphere assays further confirmed the reduced CSC activity of WTB DLL1-KD2 and DLL1-KD4 luminal cells compared to control cells (Supplementary Fig. S7D). These studies strongly suggest a supporting function of Dll1 in TIC/CSC number and activity in ERα^+^ luminal breast cancer.

### Estrogen signaling stabilizes DLL1 protein levels in luminal breast cancer

**Fig. 7:**
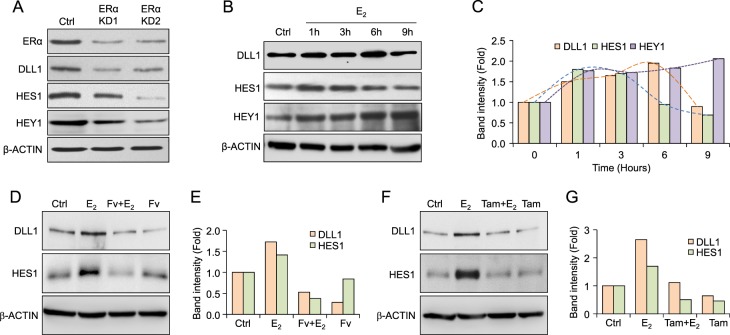
E_2_/ERα signaling enhances DLL1 protein levels. **a** Western blot shows ERα, DLL1, HES1 and HEY1 protein levels after knockdown (KD1 and KD2) of ERα in luminal MCF7 cells. **b** Western blots show protein levels of DLL1, HES1, and HEY1 after E_2_ treatment for indicated time points, which is quantified in (**c**). **d** Western blot shows protein levels of DLL1 and HES1 after indicated treatments with E_2_ and Fulvestrant (Fv) for 12 h to MCF7 cells, which is quantified in (**e**). **f** Western blot shows protein levels of DLL1 and HES1 after indicated treatments with E_2_ and Tamoxifen (Tam) for 12 h to MCF7 cells, which is quantified in (**g**)

We have shown that higher *DLL1* levels correlate with poor prognosis in ERα^+^ luminal tumors (Fig. 1c) and that high DLL1 drives ERα^+^, but not ERα^−^ tumor growth, progression, and metastasis (Figs. 2−4). These data suggest a functional contribution of ERα signaling in DLL1-mediated protumor activities. To experimentally test whether ERα signaling regulates DLL1 expression, we transiently knocked down ERα expression in MCF7 cells using shRNAs against ERα. We confirmed the knockdown of ERα protein expression in shRNA-treated cells compared to control (Fig. 7a). Interestingly, ERα knockdown led to a significant decrease in the levels of DLL1 protein but not mRNA (Fig. 7a and Supplementary Fig. S8A), indicating that the loss of ERα may reduce DLL1 protein levels by a posttranscriptional mechanism. Moreover, we found a significant decrease in HES1 and HEY1, both are known downstream targets of Notch signaling, suggesting decreased Notch signaling when ERα is reduced (Fig. 7a). To further understand the function of ERα signaling on DLL1 protein levels, we treated MCF7 cells with E_2_ (17-beta-estradiol) in charcoal-stripped serum containing medium. We found that E_2_ induced expression of DLL1 protein over time in MCF7 cells (Fig. 7b, c). This increase in DLL1 protein was also associated with increased Notch signaling as evident by increased HES1 and HEY1 expressions (Fig. 7b, c). Interestingly, increased DLL1 and HES1 expression followed a dynamic pattern in which a transient increase was followed by a drop in expression, which mimics the recently reported stochastic stereotyped pulses of DLL1-mediated Notch activation [46]. Consistent with earlier data, the increase in DLL1 protein expression was not associated with an increase in mRNA expression, highlighting the posttranscriptional role of E_2_/ERα signaling on enhancing DLL1 protein levels (Supplementary Fig. S8B). E_2_ treatment of an additional luminal breast cancer cell line, T47D showed a similar increase in DLL1 expression over time (Supplementary Fig. S8C, D). Furthermore, treatment with ERα signaling antagonists like Fulvestrant (Fv) and Tamoxifen reduced E_2_-induced DLL1 and HES1 protein expression but not DLL1 mRNA levels (Fig. 7d−g; data not shown). Notably, use of tamoxifen alone (without E_2_ treatment) reduces DLL1 and HES1 levels similar to control but not with Fulvestrant alone at 12 h of treatment, suggesting some inhibitory action of these ER antagonists on base level of DLL1 expression. As expected, E_2_ treatment of the TNBC cell lines (ER^−^) LM2 and HCC1806 did not induce DLL1 protein expression (Supplementary Fig. S8E, F), supporting the estrogen signaling dependence of DLL1 protein expression in ERα^+^ luminal tumors. Together, our data show that estrogen signaling specifically regulates DLL1 protein levels by posttranscriptional regulation in ERα^+^ luminal tumors.

### Estrogen signaling inhibits ubiquitination and proteosomal/lysosomal degradation of DLL1 in luminal tumors

**Fig. 8:**
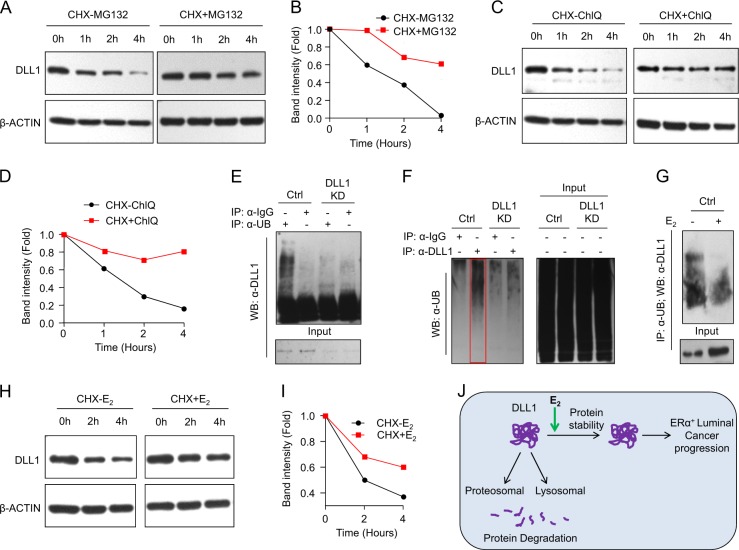
E2/ERα signaling stabilizes DLL1 protein levels from proteosomal and lysosomal degradation. **a**, **b** Western blot (**a**) shows decreased DLL1 protein levels in MCF7 cells after blocking of protein synthesis using cyclohexamide (CHX) at indicated timepoints, which is reversed with the treatment of MG132, a proteosomal inhibitor. Quantification of bands was done using ImageJ and is shown in (**b**). **c**, **d** Western blot shows decreased DLL1 protein levels in MCF7 cells after blocking of protein synthesis using cyclohexamide (CHX) at indicated timepoints, which is reversed with the treatment of Chloroquinone (ChlQ), a lysosomal inhibitor. Quantification of bands was done using ImageJ and is shown in (**d**). **e**, **f** Western blot shows polyubiquitination of endogenous DLL1 protein in hormone-deprived MCF7 cells. MCF7 (control and DLL1 KD1) cells were subjected to endogenous immune-precipitation (IP) with anti-Ubiquitin or anti-DLL1 antibodies followed with western blot using either anti-DLL1 or anti-Ubiquitin-specific antibodies respectively. Respective IgG controls were used as a negative control for IP. **g** Western blot shows decreased polyubiquitination of endogenous DLL1 protein upon 6 h of E_2_ treatment to MCF7 cells. MCF7 cells were subjected to endogenous immune-precipitation (IP) with anti-Ubiquitin antibody followed with western blot using anti-DLL1 antibody. **h**, **i** Western blot (**h**) and quantification (**i**) of DLL1 protein levels after blocking of protein synthesis using cyclohexamide (CHX) with or without E2 treatment at indicated timepoints in MCF7 cells. Quantification of bands was done using ImageJ software. **j** Schematic showing DLL1 protein degradation happens through proteosomal and lysosomal degradation, which is prevented by Estrogen signaling through E_2_/ERα in luminal breast cancer for promotion and progression of the luminal breast cancer

Protein levels depend upon the rate of protein synthesis and degradation. Protein degradation occurs largely through two pathways, the ubiquitin-proteasome and the autophagy-lysosome systems [47]. Under conditions in which the protein synthesis was blocked by treatment with cyclohexamide (CHX), we analyzed the turnover of DLL1 and observed that DLL1 exhibited a half-life of ~1.25 h (Fig. 8a, b). This half-life increased to more than 4 h upon treatment of cells with either proteasomal inhibitor (MG132, Fig. 8a, b) or with an inhibitor of the lysosomal pathway (cholorquine, Fig. 8c, d) indicating that both these pathways are likely to be involved in DLL1 degradation. Given that recruitment of proteins to proteasomes as well as endocytosis of membrane-bound proteins and their targeting to the lysosomes often relies on the conjugation of target protein with ubiquitin [48], we next examined whether DLL1 undergoes ubiquitination and the effect of E_2_ treatment on this process. Western blot analysis detected a distinct anti-DLL1 antibody-reactive smear in the immunoprecipitate of the ubiquitinated proteins (Fig. 8e), suggesting that DLL1 can undergo ubiquitination in MCF7 cells. Indeed, analysis of a reciprocal immunoprecipitation (where we pulled down the endogenous DLL1) revealed the presence of high molecular weight smears reactive to anti-Ubiquitin antibody (Fig. 8f), indicative of polyubiquitination. Importantly, these smears were less evident upon DLL1 knockdown in MCF7 cells (Fig. 8e, f), indicating the specificity of reactions in both approaches. Finally, we found that the presence of anti-DLL1 antibody-reactive smear in the immunoprecipitate of the ubiquitinated proteins could be reduced by treatment of ERα^+^ MCF7 cells with E_2_ (Fig. 8g), indicating estrogen-mediated inhibition of ubiquitination of DLL1 protein. These results collectively suggest that endogenous DLL1 undergoes ubiquitination in MCF7 cells, which could be inhibited by estrogen.

Importantly, treatment with E_2_ notably delayed a decrease in the steady-state levels of endogenous DLL1 in CHX-treated MCF7 cells (Fig. 8h, i). Given that this result has been achieved under conditions where protein synthesis is blocked by CHX, it is likely that E_2_ interfered with the rate of DLL1 turnover. Indeed, the half-life of DLL1 in E_2_-treated cells increased up to 4 h (Fig. 8h, i). These data suggest that E2 stabilizes DLL1 protein and this stabilization can, at least in part, account for the induction of DLL1 upon estrogen exposure in ERα^+^ luminal breast cancer cells.

## Discussion

Notch signaling drives many cellular processes in breast cancer [49] and the ability of GSIs, the most widely studied small molecule inhibitors of Notch signaling, to reduce breast cancer tumor growth [21] identifies Notch signaling as an attractive therapeutic target for breast cancer. Unfortunately, GSIs target all Notch signaling plus other gamma-secretase-dependent signals and are not well tolerated by patients. Thus, a therapeutic approach that specifically targets unique receptor- or ligand-driven Notch signaling pathways that drive tumor cell progression may provide a safer and more effective alternative for the treatment of breast cancer. While several studies have described the involvement of different Notch receptors in breast cancer, a detailed analysis of the ligands regulating breast cancer initiation, progression, and metastasis is lacking. In this study, we identify the Notch ligand DLL1 as a potential therapeutic target for ERα^+^ luminal breast cancer.

Using a Dll1 antibody, we now show that DLL1 protein is specifically overexpressed in ERα^+^ luminal breast cancer patient samples when compared to normal tissue or TNBC tumors. Moreover, higher *DLL1* expression is specifically associated with poor prognosis in ERα^+^ luminal A breast cancer patients and expression levels of most other Notch ligands did not reflect prognosis. We did observe a correlation with *DLL4* and poor prognosis in luminal A breast cancer patients. It is possible that DLL4 may have tumor-promoting function in luminal breast cancer, which needs future careful evaluation. When *DLL1* was stratified into high and low in Luminal B patients, modest trend was observed which did not receive significance due to the fact that Luminal A patients have higher ERα expression. In contrast, high *DLL1* expression was associated with enhanced survival of TNBC/basal breast cancer patients. Notably, these findings were further confirmed by DLL1 protein expression data in which a correlation between higher DLL1 protein levels and poor overall survival was observed in the luminal subset. Corroborating our clinical data, our functional data using mouse and human breast cancer cell lines strongly support a protumorigenic role for Dll1 in ERα^+^ luminal breast cancer (Figs. 2–4). Interestingly, Dll1 promotes both primary tumor growth and lung metastasis of the luminal subtype of breast cancer, while it has little or no effect on tumor growth of TNBC. It is possible that DLL1 may have a metastasis suppressor effect on TNBC cells, which needs further exploration. Recently, our group showed that Dll1^+^ cells crosstalk with stromal cells such as macrophages to promote mammary stem function in normal breast [28]. It is possible that DLL1 has opposing functions in breast cancer subtypes due to its interaction with distinct cell types within the different tumor microenvironments of different subtypes, which needs further evaluation. Together, our data reveal for the first time the subtype-specific function of DLL1 in promoting tumor growth, progression, and metastasis of ERα^+^ luminal breast cancer.

Notch signaling promotes proliferation and increases CSC activity. Our data suggest a supporting role for DLL1 in promoting tumor cell proliferation and angiogenesis. All these factors play an essential role in tumor growth and progression. Recent studies showed that DLL1 is associated with the CSC activity of glioblastoma and renal cell carcinoma, rhabdomyosarcoma [29–31]. Our data show that loss of DLL1 reduces CSC number and function in luminal breast cancer cells. While many studies indicate that basal/TNBC breast cancer is associated with increased cancer stem cells [50], the presence of CSCs in luminal breast cancer is poorly understood. One recent study implicated that decreased Notch signaling increases CSCs and treatment resistance in MCF7 cells [51]; however, the mechanism was not well understood. Importantly, our data indicate that the presence of CSCs in tamoxifen-sensitive ERα^+^ luminal breast cancer is dependent on DLL1-mediated Notch signaling. This result is consistent with importance of DLL1 in mammary cell stemness as reported by our recent study regarding the role of Dll1 in the function of the normal mammary stem cells [28]. We speculate that the mode of ligand-specified Notch signaling is different between tamoxifen-sensitive and tamoxifen-resistant breast cancers; this suggestion needs to be carefully evaluated in the future studies. As CSCs are well known to be associated with chemotherapy and radiation resistance, ongoing studies are aimed to explore the possibility of a potential therapeutic benefit of targeting DLL1 to reduce the number of CSCs in treatment-resistant ERα^+^ tumors.

Mechanistically, we found that DLL1 protein is poly-ubiquitinated and undergoes proteasomal/lysosomal degradation in untreated ERα^+^ luminal cells. Importantly, estrogen signaling interferes with DLL1 turnover and therefore stabilizes DLL1 protein (Fig. 8) leading to an induction of its steady-state levels (Fig. 7). While our results suggest that DLL1 is posttranscriptionally regulated, we do not want to rule out the possibility that DLL1 may also be regulated transcriptionally (either estrogen-dependent or estrogen-independent), a possibility that will require further exploration. Studies have shown that Notch receptors undergo rapid degradation through a ubiquitin-mediated proteasomal pathway [52, 53]; however, little is known regarding the mechanisms that regulate stability and abundance of Notch ligands and the role of these mechanisms in cancer. Thus, our study shows for the first time a unique molecular mechanism by which DLL1 ligand of Notch pathway is regulated during breast cancer. Future ongoing studies in the laboratory will explore which E3 ligases may be responsible for ubiquitination of DLL1 and how the activities of these ligases may be impeded by estrogens. Notably, an estrogen-induced activation of Notch signaling has been seen recently in ERα^+^ endometrial cancer [54], although the mechanistic basis of the crosstalk between estrogen and Notch remains controversial [55, 56]. In our hands, we find that estrogen-induced stabilization of DLL1 protein may facilitate luminal cancer progression with important implications for the treatment of ERα^+^ breast cancer patients. Specifically, there is accumulating evidence to suggest that an increase in breast CSCs occurs following endocrine therapy of ERα^+^ tumors. It is possible that estrogen-enhanced DLL1-dependent Notch signaling increases the abundance of CSCs populations in these patients. Future studies will delineate if resistance and relapse in ERα^+^ breast cancers is dependent on DLL1-mediated Notch signaling, as such studies would aid in developing novel therapies for ERα^+^ luminal breast cancer patients.

Overall, our study demonstrates for the first time that the Notch ligand DLL1 is overexpressed in ERα^+^ luminal breast cancer. Furthermore, our data indicate that DLL1-mediated Notch signaling is regulated by estrogen signaling at the posttranscriptional level to stabilize DLL1 protein (Fig. 8j). As a consequence, enhanced DLL1-driven Notch signaling serves as a driving force for the initiation, progression, and metastasis of luminal breast tumor (Fig. 8j). Together, our findings highlight a novel subtype-specific function of DLL1-driven Notch signaling as a potential target for therapeutic intervention for this large subset of breast cancer patients.

## Materials and methods

### Human patient samples

Normal human breast tissues and breast cancer specimens used in the study were de-identified samples and were obtained from The Eastern Division of the Cooperative Human Tissue Network (CHTN), University of Pennsylvania and in collaboration with Dr. Qing Zhang at the University of North Carolina (Supplementary Table S1A, B). All samples were considered exempt by Institutional Review Board of University of Pennsylvania and the University of North Carolina at Chapel Hill. DLL1 antibody (Abcam #ab84620) was applied at a dilution of 1:60 and incubated at 4 °C for overnight. All DLL1-stained IHC samples were scored for both intensity of DLL1 protein expression and abundance of DLL1^+^ cells. The intensity of the DLL1 protein was measured using the scale 0–3, 0 being negative and 3 being very high expression. The abundance of positive cells in the tissue was measured using a scale ranging from 0 to 100. The intensity score of more than 1 was considered to be positive cells to score abundance. The H-Score was calculated by multiplying intensity with abundance. Mann−Whitney *U* test was performed to assess statistical significance.

### Cell culture studies

Human breast cancer cell lines MCF7, T47D, LM2, Hs578T, and BT549 were originally obtained from American Type Culture Collection (ATCC). Human luminal ZR-75-1 cell line was purchased from Sigma-Aldrich. Mouse WTB, 4T1, and human SUM159 were a kind gift from Dr. Yibin Kang’s laboratory, Princeton University, NJ. HCC1806 was a kind gift from Dr. Sophie Ran’s laboratory, Southern Illinois University School of Medicine, IL. MCF7, HCC1806, LM2 (lung derivative of MDA-MB–231), WTB, 4T1, Hs579T cell lines were grown in Dulbecco’s modified Eagle’s medium (DMEM) (Sigma-Aldrich) and T47D, BT549, ZR-75-1 cell lines were grown in RPMI medium, and SUM159 cell line was grown in F12 medium. All cells were supplemented with 10% fetal bovine serum (FBS) (except HCC1806 with 5% FBS) (Invitrogen), 100 U/ml Penicillin and 100 µg/ml Streptomycin sulfate (Invitrogen). MCF7 and 4T1 cell lines were supplemented with 10 μg/ml insulin. All the cell lines were maintained at 37 °C in a humidified atmosphere containing 5% CO_2_ in the incubator. For estrogen-related studies, breast cancer cells (MCF7, T47D, LM2, and HCC1806) were cultured in Phenol red-free DMEM containing 2–5% charcoal-stripped FBS for 48 h prior to treatment with 17β-estradiol (E_2_) 100 nM, fulvestrant 200 nM or tamoxifen 200 nM. Cyclohexamide, MG132 or lysosomal inhibitors chloroquinone were used at a final concentration of 50 µg/ml, 1 µM and 100 µM respectively. All cells were tested and confirmed to be mycoplasma negative. Human breast cancer cells were authenticated by STR DNA profiling analysis.

### Viral production and infectionere

For lentivirus-mediated knockdown studies, shRNA constructs (SHCLNG-NM_005618 and SHCLNG-NM_007865) were obtained from Sigma-Aldrich. Empty pLKO.1 vector from Sigma-Aldrich was used as control as backbone vector for all shRNAs was the empty pLKO.1 vector. We used previously described Dll1 overexpression construct, in which Dll1 cDNA was cloned into pLEX MCS plasmid (Open Biosystems) [28]. All plasmids were packaged into virus using HEK293-T cells as packaging cell lines and helper plasmids VSVG and dR8.9 following standard protocols [57, 58]. For ERα shRNA constructs, transfection was performed using Lipofectamine 2000. Constructs were purchased and validated from Origene (Cat #TR320346). All breast cancer cell lines were plated and infected with virus-containing media supplemented [28] with 2 μg/ml polybrene for 24−48 h. After infection, media were replaced with fresh media containing puromycin (1–3 μg/ml) for selection of the virus-infected cells.

### Animal studies

Animal procedures were conducted in compliance with Institutional Animal Care and Use Committee (IACUC) of University of Pennsylvania. FVB, NSG, and BALB/c mice were obtained from Jackson Laboratory. For mammary fat pad injection experiments, all mice at 5–7 weeks old were anaesthetized and tumor cells were injected into the mammary fat pad following established protocols [59]. For MCF7 cells, mice were supplemented with estradiol tablets, which were placed subcutaneously in the dorsal neck region of mice in each group. After mammary fat pad injection, the mice were weighed and each mammary gland tumor was palpated manually and measured with digital caliper once a week until sacrifice. Tumor size was assessed by external measurement of length (*L*) and width (*W*) of tumors. The tumor volume (mm^3^) was calculated by using the following equation: tumor volume = ((*π* *L* × *W*^2^)/6). For intravenous injection, mice were injected with tumor cells using established protocol [59]. During experiment, the animals were closely monitored for any signs of morbidity, declining body weight, ruffled fur, hunched posture, and ulceration of tumor. At the experimental end point, all mice were euthanized by cervical dislocation to harvest primary tumors and metastatic organs for further analysis.

### Lung nodule count

Tumor-bearing mice were sacrificed by cervical dislocation method to harvest the metastatic lungs. The lungs were immediately washed twice with PBS and fixed in Bouin’s fixative for 24 h. The fixed lungs were twice washed with 70% ethanol after fixation. The number of lung nodules was counted with the aid of a dissecting microscope. Lungs were then processed and embedded in paraffin and sectioned followed by H&E staining for further evaluation of lung area, number etc. The lung nodule area from sections stained with H&E was measured using ImageJ.

### Bioluminescence imaging of tumors and lung tissues

Mice were anesthetized with ketamine/xylazine and were injected retro-orbitally with 100 μl of Firefly d-Luciferin substrate (Gold Biotechnology, 15 mg/ml) for labeled MCF7 cells. Bioluminescence images of in vivo tumor and lungs, as well as harvested lung tissues, were acquired 5 min after injection of d-Luciferin using the IVIS Spectrum system (Caliper Life Sciences). Data are expressed as total photon flux and were analyzed using Living Image 3.0 software (Caliper Life Sciences).

### Tumorsphere assay

One thousand tumor cells were cultured as previously described [58]. Briefly, cells were cultured in low adherent plates and were grown in serum-free mammary epithelial media from LONZA supplemented with B27 (1×, Invitrogen), Epidermal growth factor (EGF) (20 ng/ml) and Fibroblast growth factor (FGF) (20 ng/ml).

### Protein extraction, immunoprecipitation, and western blot analysis

Proteins were extracted from primary epithelial cell cultures and cell lines in RIPA buffer as previously described [60, 61]. For immunoprecipitation, cells were suspended in 1% triton-X100 containing Phosphate-Buffered Saline (PBS) and Sodium dodecyl Sulfate (SDS). Cell lysate (4 mg total protein) was made by boiling samples at 100 °C followed by sonication. Lysate was precleared by incubating with 50 µl of protein A+G agarose beads (Fisher Scientific) for 1 h at 4 °C. 2 µg of Anti-Ubiquitin antibody (Santa Cruz, sc#8017) or anti-DLL1 antibody (Abcam, ab84620) or respective species specific control antibodies were incubated overnight with 4 mg of proteins. At the time of antibody addition, complete protease inhibitor and phosphatase inhibitors (Roche) were added besides 1 mM Dithiothreitol (DTT). Lysates were incubated overnight with the antibodies followed by three washes with triton-X100 containing PBS and one wash with 20% sucrose in PBS. Western blot analysis was performed using the standard protocol.

### Histological analysis, immunohistochemistry, and immunofluorescence

For histological analysis, mammary tumor specimens were processed as previously described [60, 61]. Antibodies and dilutions used are listed in the Supplementary Table S2. 4′,6-diamidino-2-phenylindole (DAPI) was used to stain nuclei. Images were taken using Nikon TiE microscope. Scoring was done by examining multiple fields of view (FOV) per sample.

### Flow cytometry

The single-cell suspension of tumors from WTB and MCF7 cells was obtained following published protocol [58]. WTB and MCF7 control and DLL1-KD cells in culture were trypsinized for single-cell suspension. The single cells obtained from tumor tissues or cell lines were stained with a combination of antibodies (listed in the Supplementary Table S2) for 30 min on room temperature in the dark. Fluorescence-activated cell sorting (FACS) analysis was performed using the LSRII Flow Cytometer (BD Biosciences) and data were analyzed using FlowJo software (TreeStar, Inc).

### qRT-PCR analyses

Total RNA was isolated from cultured cell lines using Qiagen RNA extraction kit in accordance with the manufacturer’s instructions. Real-time RT-PCR was performed on the QuantStudio 3 Real-Time PCR system (ThermoFisher) using SYBR Green Power (Life Science Technologies). The gene-specific primer sets were used at a final concentration of 0.2 μM and their sequences are listed in Supplementary Table S3. All qRT-PCR assays were performed in duplicate in at least three independent experiments using three different tissue samples.

### Statistical analysis

Results were reported as mean ± SD (standard deviation) or mean ± SEM (standard error of the mean). The significance of differences was calculated using two-tailed Student’s *t* test for normally distributed datasets. Normal distribution of data was evaluated by Shapiro Wilk *W* test or Skewness Kurtosis test for Normality. Non-normally distributed datasets were analyzed using nonparametric Mann−Whitney *U* tests. The tumor growth datasets were analyzed using the Bonferroni corrected two-way ANOVA to compute statistical significance. Differences in survival between groups via Kaplan−Meier plots were statistically evaluated using Log-Rank tests.

## Electronic supplementary material

Supplemental figures S1-8 and tables S1-3
