# Supplementary material for: Estrogen-dependent DLL1-mediated Notch signaling promotes luminal breast cancer
Source: Oncogene. 2018 Nov 15;38(12):2092–107. doi: 10.1038/s41388-018-0562-z (PMC6756232; doi:10.1038/s41388-018-0562-z)
Supplement: Supplementary file 1 — Supplemental figures S1-8 and tables S1-3 [file 41388_2018_562_MOESM1_ESM.pdf]

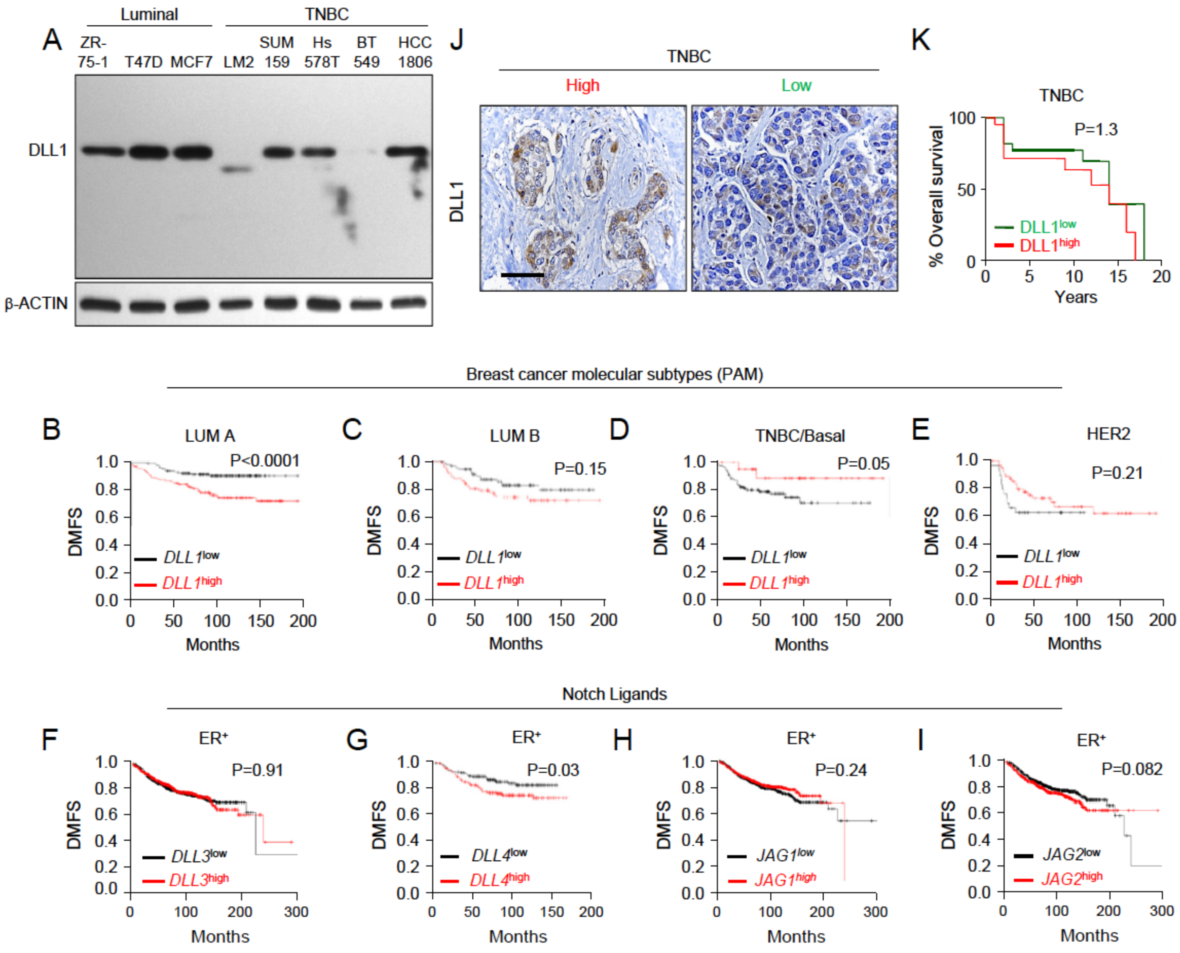

Supplementary Figure S1. Patients with ER^+ luminal tumors having high DLL1 expression is associated with poor patient survival. (**A**) Western blot showing DLL1 protein levels in different human breast cancer cell lines. (**B-E**) Kaplan–Meier plots showing poor distant metastasis free survival (DMFS) of breast cancer patients with high *DLL1* expression status in luminal A (**B**), but not in luminal B (**C**), basal/TNBC (**D**) and $HER2^+$ (**E**) subtypes. n=965 (**B**), n=430 (**C**), n=232 (**D**) and n=119 (**E**) breast cancer patients were used to make KM-plots. (**F-I**) Kaplan–Meier plots showing distant metastasis free survival (DMFS) of $ER\alpha^+$ breast cancer patients based on *DLL3* (**F**), *DLL4* (**G**), *JAGGED1* (**H**) and *JAGGED2* (**I**) expression status. n=1395 (**F**), n=437 (**G**), n=1395 (**H**) and n=1395 (**I**) ER^+ patients were used to make KM-plots. (**J**) Representative IHC images of TNBC patient breast tumors showing $DLL1^{high}$ and $DLL1^{low}$ expression respectively after staining with DLL1 antibody. (**K**) Kaplan–Meier plot is not showing any significant difference in overall survival between $DLL1^{high}$ and $DLL1^{low}$ patients in TNBC

subtype. H-score was used to stratify patients into DLL1 high and low expressers based on the IHC on the TNBC tumors with DLL1 antibody. n=21 TNBC samples were used with overall survival data. Samples are stratified into $DLL1^{low}$ (n=10) and $DLL1^{high}$ (n=11) patient groups for KM plot analysis. (**B-I, K**) Log-rank test was used for Kaplan–Meier plots to calculate p-values. Scale bars, 40μm (**I**).

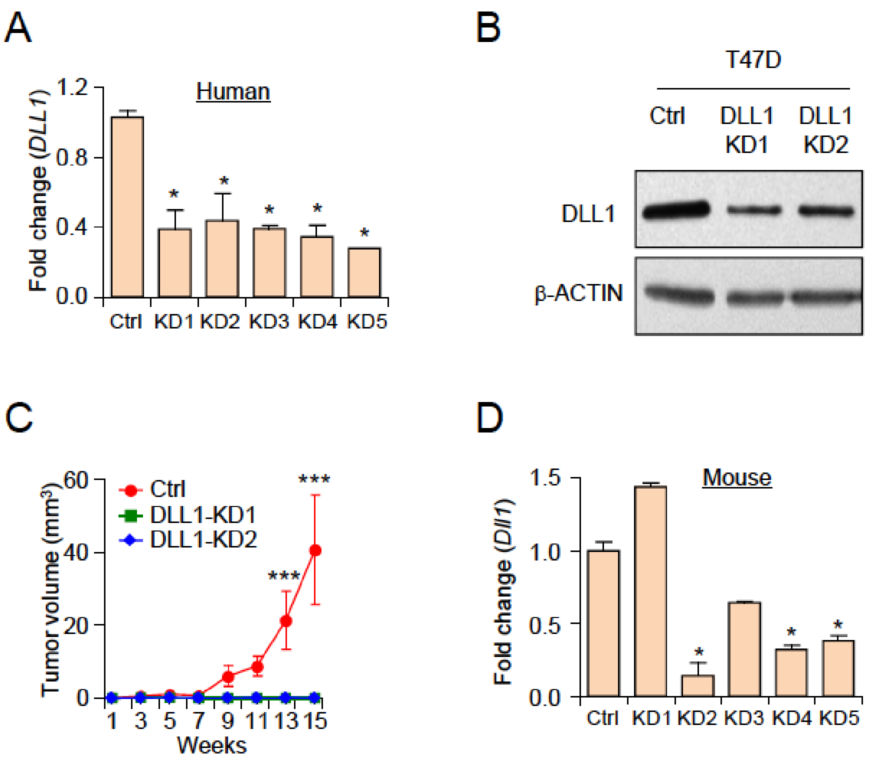

Supplementary Figure S2. Reduced level of DLL1 in luminal tumor cells inhibits tumor growth. (**A**) qPCR data showing mRNA levels of *DLL1* in human luminal breast cancer cell line T47D upon lentivirus mediated shRNA knock-down. Multiple shRNAs were tested. (**B**) Western blot data showing level of DLL1 protein in human luminal/non-TNBC cell line T47D after lentivirus mediated knock-down of DLL1 (KD). (**C**) Tumor growth curves showing growth of T47D control, DLL1-KD (KD1 and KD2) cells after injection of 2 million cells into mammary fat pad (MFP) of NSG mice. Ctrl n=8 tumors, DLL1-KD1 n=6 tumors and DLL1-KD2 n=6 tumors. (**D**) qPCR data showing mRNA levels of *Dll1* in mouse breast cancer cell line (WTB) upon lentivirus mediated knock-down of Dll1. Multiple shRNAs were tested. Student's *t*-test was used for (**A, D**). 2-way ANOVA test was performed to compute statistical significance for tumor growth curve data (**C**). Data are presented as the mean ± SEM. *p<0.05.

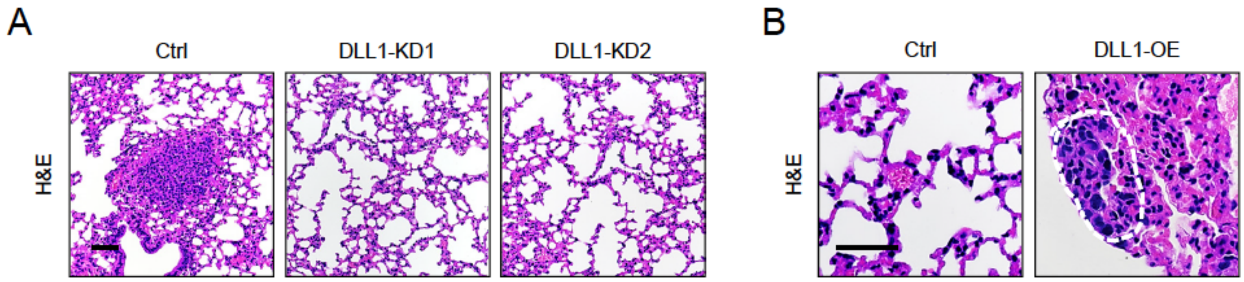

Supplementary Figure S3. DLL1 promotes metastasis in human luminal tumors. (**A**) Representative H&E images are showing reduced lung metastasis in mice injected with MCF7 DLL1-KDs (KD1 and KD2) compared to control. (**B**) Representative H&E images are showing enhanced lung metastasis in MCF7 DLL1-OE compared to control. Scale bars, 100 µm in (**A**) and 40µm (**B**) respectively.

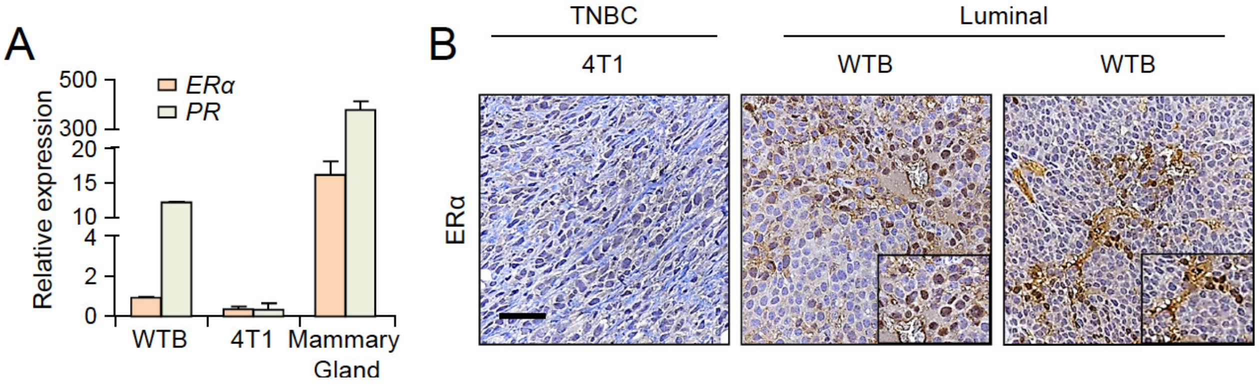

Supplementary Figure S4. Luminal WTB cells express Estrogen Receptor α (ERα) and Progesterone Receptor (PR). (**A**) qPCR data is showing mRNA level expression of ERα and PR in WTB (luminal), 4T1 (TNBC) cells and normal mouse mammary epithelial cells. WTB is derived from MMTV-PyMT luminal breast cancer model. (**B**) Representative IHC images are showing positive ERα staining in WTB (luminal) xenograft tumors compared to 4T1 (TNBC) xenograft tumors. Scale bars, 40μm (**B**). Data are presented as the mean ± SD.

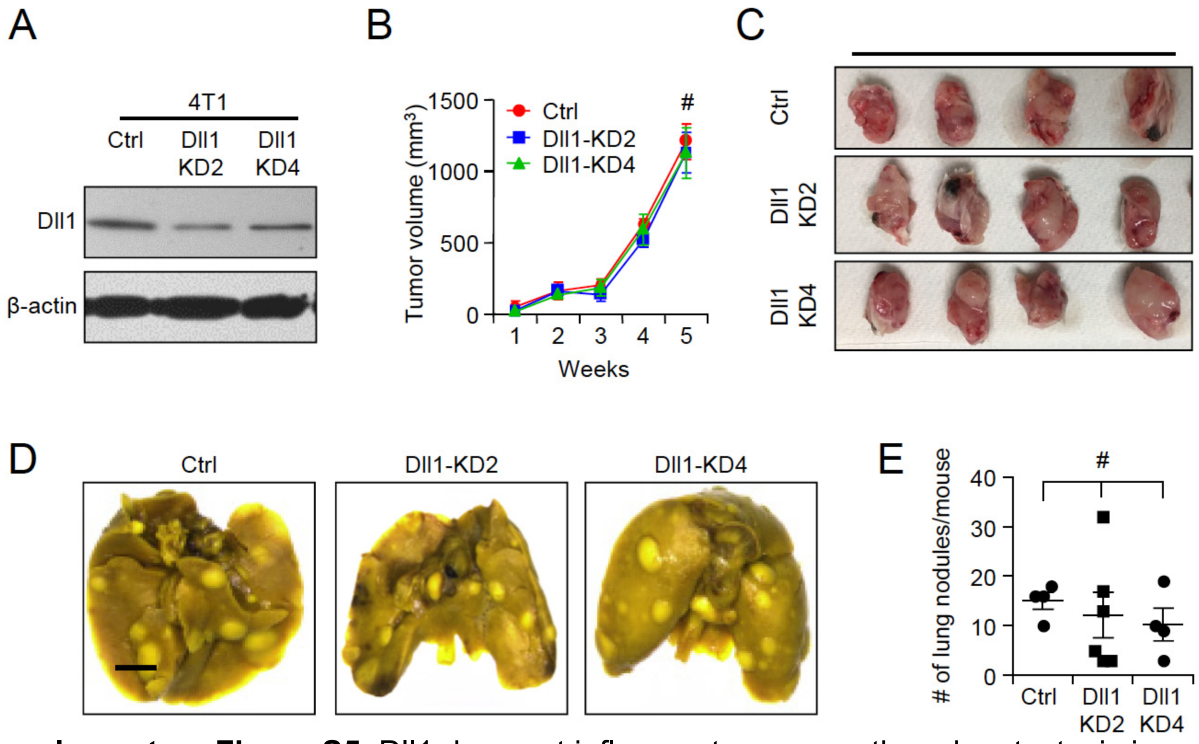

Supplementary Figure S5. Dll1 does not influence tumor growth and metastasis in mouse TNBC cells. (**A)** Western blot data is showing expression levels of Dll1 protein in mouse 4T1 cells after lentivirus mediated shRNA knock-down (KD). (**B**) 20,000 4T1 control and Dll1-KDs (KD2 and KD4) cells were injected into mammary fat pad of BALB/c mice. Tumor growth curves (**B**) and representative whole tumor images (**C**) show no significant difference in growth of 4T1 Dll1-KDs (KD2 and KD4) primary tumors compared to control. (**D**) Representative whole mount images of lungs from mice with mammary fat pad injection (MFP) showing metastasis (**D**) and quantification is shown in (**E**). Contralateral mammary glands (4th position) of Ctrl n=4, Dll1-KD2 n=6 and Dll1-KD4 n=4 mice were used for injection/group. (**E**) Mann-Whitney *U*-test and (**B**) 2-way ANOVA test was performed to compute statistical significance. Scale bars, 500μm in (**D**). Data are presented as the mean ± SEM. # non-significant.

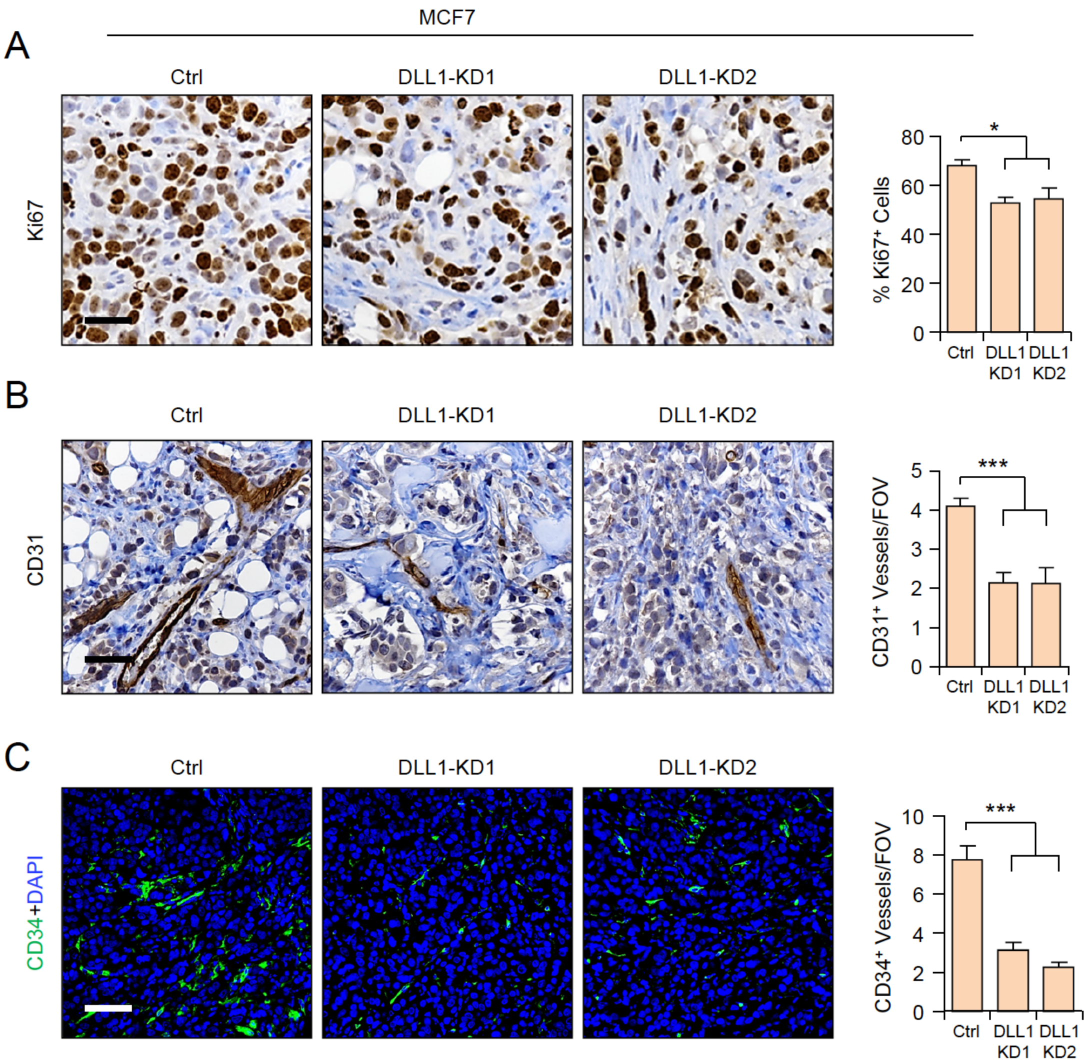

Supplementary Figure S6. Reduction of DLL1 in human ERα^{+} luminal tumors leads to decreased proliferation and angiogenesis. (**A-B**) Representative IHC images are showing reduced proliferation (% of Ki67^{+} cells) (**A**) and CD31^{+} blood vessels (**B**) in MCF7 DLL1-KDs (KD1 and KD2) primary tumors compared to control. Quantification is shown in the right of representative images. (**C**) Representative IF images are showing reduced CD34^{+} blood vessels in MCF7 DLL1-KDs (KD1 and KD2) primary tumors compared to control. Quantification is shown in the right. (**A-C**) Mann-Whitney *U*-test to compute p-values. Scale bars, 40μm (**A-C**). *p<0.05 and ***p<0.001. IHC images were quantified from 10 random fields of 3 different samples per group. FOV; field of view. Data are presented as the mean ± SEM.

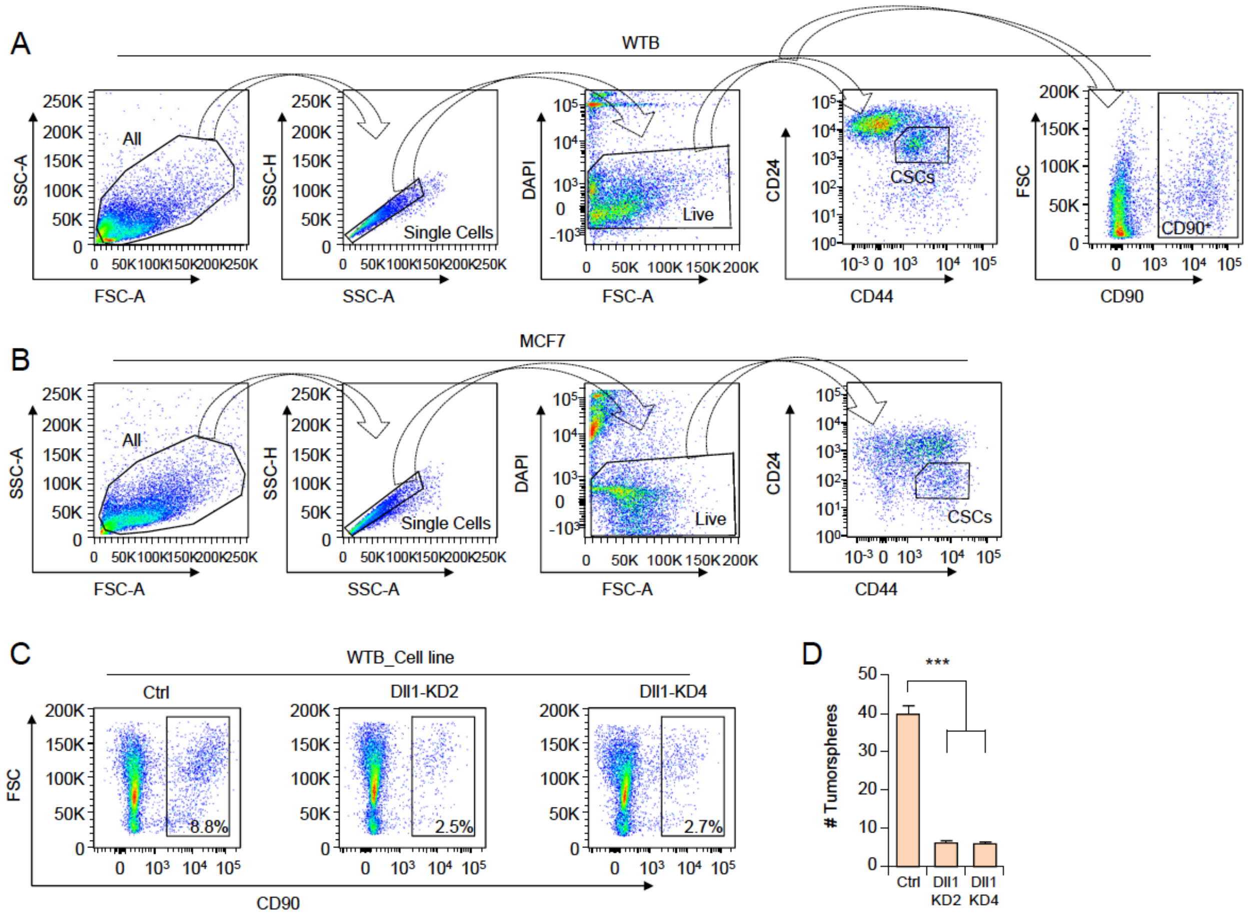

Supplementary Figure S7. Detailed gating strategy from FACS for CSC population in luminal tumors. (A, B) FACS plots are showing gating strategies to analyze and quantify the CSCs population using CD24/CD44 and CD90 CSCs markers in WTB (A) and MCF7 (B) cells and tumors. (C) FACS data show reduced in vitro CSCs population (CD90+ population) in WTB Dll1-KDs (KD2 and KD4) cells compared to control cells. (D) Bar graph is showing number of total tumorspheres in WTB control and Dll1-KDs (KD2 and KD4) cells, where 1000 cells/indicated group were cultured in the low adherent plates for 5 days in tumorsphere media. Student's t-test was used to compute p-values. Data are presented as the mean ± SEM. ***$p<0.001$.

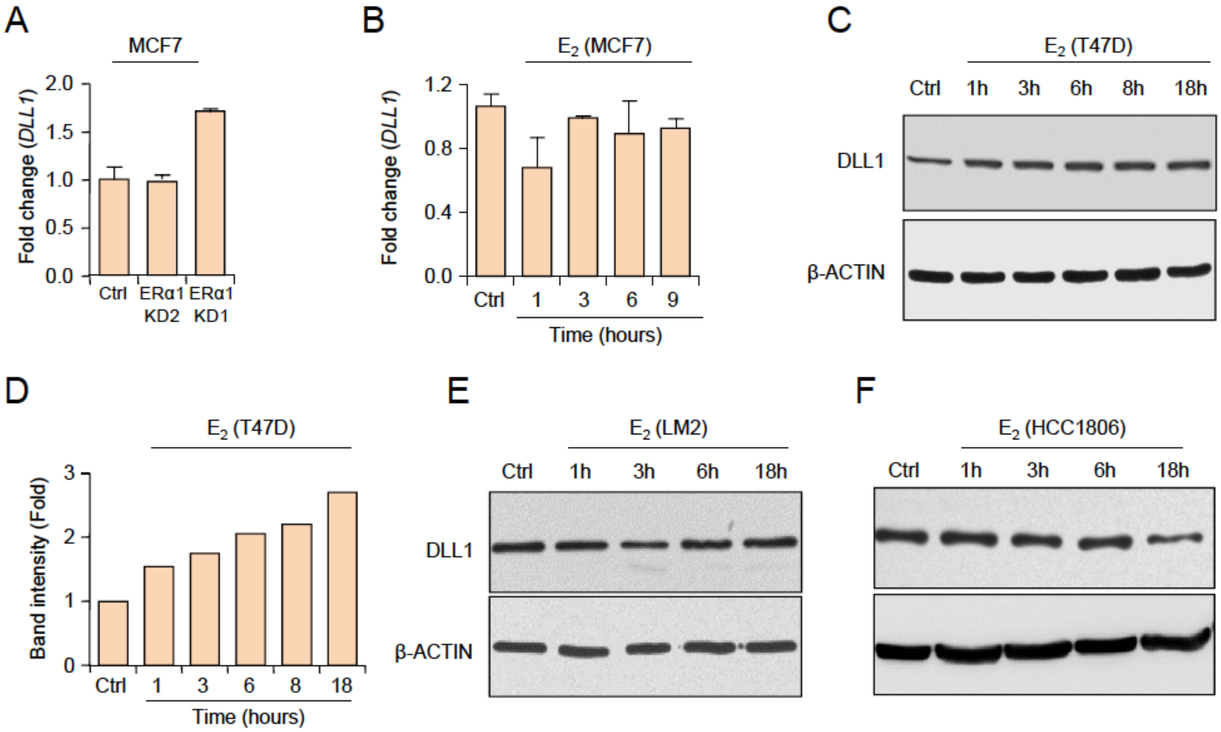

Supplementary Figure S8. Estrogen signaling alters DLL1 protein but not mRNA levels in luminal tumor cells. (**A**) qPCR analysis showing the mRNA levels of *DLL1* after shRNA mediated knock down of ERα in MCF7 cells. (**B**) qPCR analysis showing mRNA levels of *DLL1* upon E_2 treatment for indicated time points. (**C, E-F**) Western blots are showing protein levels of DLL1 after E_2 treatment for indicated time points in T47D (**C**), LM2 (**E**) and HCC1806 (**F**) cells. (**D**) Bar graph is showing the quantification of the western blot bands shown in **C**. Data are presented as the mean ± SD in (**A**, **B**).

Supplementary table S1A: ER/PR/HER2 status on patients

Non-TNBC patients

**Identifier**	**ER**	**PR**	**HER2**
51062T	Positive	Positive	Negative
56455T	Positive	Positive	Positive
57113T	Positive	Positive	Positive
57426T	Positive	Positive	Positive
61734T	Positive	Positive	Negative
65657T	Positive	Positive	Positive
67551T	Positive	Positive	Positive
69936T	Positive	Positive	Positive
64973T	Positive	Positive	Positive
68490T	Positive	Positive	Negative
68831T	Positive	Negative	Negative
69141T	Positive	Negative	Negative
70109T	Positive	Positive	Negative
68477T	Positive	Positive	Negative
68510T	Positive	Positive	Negative
67604T	Positive	Positive	Negative
67201T	Positive	Positive	Negative
77074T	Positive	Positive	Negative
57038T	Positive	Positive	Negative
67316T	Positive	Positive	Negative
67710T	Positive	Positive	Negative
060028	Positive	Negative	Negative
060756	Positive	Positive	Positive
061398	Positive	Negative	Positive
070158	Positive	Positive	Positive
072675	Positive	Negative	Negative
080846	Positive	Positive	Positive
081996	Positive	Positive	Positive
082333	Positive	Negative	Negative
090070	Positive	Positive	Positive
090128	Positive	Positive	Positive

090813	Positive	Positive	Negative
101007	Positive	Positive	Positive
101126	Positive	Negative	Positive
101187	Positive	Positive	Negative
111332	Positive	Positive	Negative
121508	Positive	Negative	Positive
121555	Positive	Negative	Positive
130236	Positive	Negative	Negative
060490	Positive	Positive	Negative
050387	Positive	Negative	Negative
050682	Positive	Positive	Negative
050076	Positive	Positive	Negative
040297	Positive	Positive	Negative
040114	Positive	Positive	Positive
030468	Positive	Positive	Negative
010419	Positive	Negative	Negative
010413	Positive	Negative	Positive
61413	Positive	Positive	Negative
71403	Positive	Positive	Negative
71340	Positive	Positive	Negative
40211	Positive	Negative	Negative
71458	Positive	Positive	Negative
70804	Positive	Positive	Negative
72572	Positive	Positive	Negative
60121	Positive	Positive	Negative
60678	Positive	Positive	Positive
70732	Positive	Positive	Positive
72677	Positive	Positive	Negative
71997	Positive	Positive	Positive

TNBC patients

Identifier	ER	PR	HER2
57015T	Negative	Negative	Negative
58380T	Negative	Negative	Negative
63407T	Negative	Negative	Negative
63998T	Negative	Negative	Negative
63997T	Negative	Negative	Negative
63999T	Negative	Negative	Negative
69221T	Negative	Negative	Negative
75275T	Negative	Negative	Negative
56393T	Negative	Negative	Negative
69144T	Negative	Negative	Negative
69231T	Negative	Negative	Negative
77241T	Negative	Negative	Negative
68168T	Negative	Negative	Negative
65721T	Negative	Negative	Negative
69227T	N/A	N/A	N/A
70205T	Negative	Negative	Negative
67190T	Positive	Negative	Negative
68859t	Negative	Negative	Negative
68892T	Negative	Negative	Negative
69923T	Negative	Negative	Negative
69924T	Negative	Negative	Negative/Equivocal
69927T	Negative	Negative	Negative
50759	Negative	Negative	Negative
61093	Negative	Negative	Negative
70594	Negative	Negative	Negative
70726	Negative	Negative	Negative
71152	Negative	Negative	Negative
71805	Negative	Negative	Negative
80307	Negative	Negative	Negative
80478	Negative	Negative	Negative
80912	Negative	Negative	Negative
81785	Negative	Negative	Negative
82366	Negative	Negative	Negative
82383	Negative	Negative	Negative
111162	Negative	Negative	Negative
120946	Negative	Negative	Negative
121427	Negative	Negative	Negative
140133	Negative	Negative	Negative
10049	Negative	Negative	Negative
10208	Negative	Negative	Negative
10532	Negative	Negative	Negative
30078	Negative	Negative	Negative
30408	Negative	Negative	Positive
40371	Negative	Negative	Negative
40402	Negative	Negative	Negative
40415	Negative	Negative	Negative

40593	Negative	Negative	Negative
50259	Negative	Negative	Negative
50362	Negative	Negative	Negative
60952	Negative	Negative	Negative
80206	Negative	Negative	Negative
990141	Negative	Negative	Negative
101412	Negative	Negative	Negative
110160	Negative	Negative	Negative
50285	Negative	Negative	Negative
70331	Negative	Negative	Negative
10353	Negative	Negative	Negative

Supplementary table S1B: ER/PR/HER2 status on patients

Non-TNBC patient for overall survival analysis:

Identifier	ER	PR	HER2
10413	Positive	Negative	Positive
71403	Positive	Positive	Negative
70804	Positive	Positive	Negative
72572	Positive	Positive	Negative
50076	Positive	Positive	Negative
40114	Positive	Positive	Positive
072675	Positive	Negative	Negative
080846	Positive	Positive	Positive
090128	Positive	Positive	Positive
090813	Positive	Positive	Negative
101126	Positive	Negative	Positive
101007	Positive	Positive	Positive
60678	Positive	Positive	Positive
40297	Positive	Positive	Negative
50387	Positive	Negative	Negative
70732	Positive	Positive	Positive
71997	Positive	Positive	Positive
60490	Positive	Positive	Negative
50682	Positive	Positive	Negative
71340	Positive	Positive	Negative
71458	Positive	Positive	Negative
60121	Positive	Positive	Negative
060756	Positive	Positive	Positive
061398	Positive	Negative	Positive
72677	Positive	Positive	Negative
081996	Positive	Positive	Positive
082333	Positive	Negative	Negative
090070	Positive	Positive	Positive
101187	Positive	Positive	Negative
121508	Positive	Negative	Positive
121555	Positive	Negative	Positive
130236	Positive	Negative	Negative

TNBC patient for overall survival analysis:

Identifier	ER	PR	HER2
10532	Negative	Negative	Negative
80478	Negative	Negative	Negative
140133	Negative	Negative	Negative
40402	Negative	Negative	Negative
50362	Negative	Negative	Negative
80206	Negative	Negative	Negative
50259	Negative	Negative	Negative
40415	Negative	Negative	Negative
30408	Negative	Negative	Positive
10208	Negative	Negative	Negative
990141	Negative	Negative	Negative
110160	Negative	Negative	Negative
40593	Negative	Negative	Negative
50285	Negative	Negative	Negative
70331	Negative	Negative	Negative
101412	Negative	Negative	Negative
10049	Negative	Negative	Negative
60952	Negative	Negative	Negative
40371	Negative	Negative	Negative
30078	Negative	Negative	Negative
10353	Negative	Negative	Negative

Supplementary table S2: List of antibodies used.

Flow cytometry

Antibody	Fluorochrome	Dilution	Company
CD24 (hu)	BV421	1:100	BD Biosciences, Cat #562789
CD24 (mo)	BV421	1:100	BD Biosciences, Cat #562563
CD44 (hu, mo)	PE-Cy7	1:100	Biolegend, Cat# 103030
CD90 (hu)	APC	1:100	eBioscience, Cat #17-0909-41
CD90 (mo)	FITC	1:100	eBioscience, Cat #11-0900-81

Western blot/IHC/IF

Antibody	IHC	IF	WB	Company
IgG	1:100	-	-	Cell Signaling Tech., Cat # 2729S
DLL1	1:60	-	1:1000	Abcam, Cat # ab84620
β-actin	-	-	1:10,000	Abcam, Cat #ab8227
Ki67	1:50	-	-	Novocastra, Cat # NC9025734
CD31	1:100	-	-	Abcam, Cat #ab28364
HES1	-	-	1:1000	Abcam, Cat # ab71559
CD34	-	1:100	-	Abcam, Cat # ab81289
ERα	1:75	-	1:1000	Thermo Fisher, Cat # MA112692
HEY1	-	-	1:1000	Millipore Sigma, Cat # AB5714

Supplementary table S3: Primers used for quantitative PCR

Gene	Forward	Reverse
hDLL1	GCAGCTCTTCACCCTGTTCT	GGTGCAGGAGAAGTCGTTCA
hGAPDH	GGAGTCAACGGATTTGGTCGTA	GGCAACAATATCCACTTTACCAGAGT
mDll1	GCGAGCTGCACGGACCTTGA	GCCCAAGGGGCAATGGCAGG
mERα	AATGCAAGAACGTTGTGCCC	TCTGCTTCCGGGGGTATGTA
mPR	GTGTCGTCTGTAGTCTCGCC	GAGAAAGCTCCCTCCACGTC
mGapdh	TTCCACTCTTCCACCTTCGATGC	GGGTCTGGGATGGAAATTGTGAGG
